# Multiplexing Two Types of High‐Order Topological Corner States in Kagome Lattice Photonic Crystals

**DOI:** 10.1002/nap2.70095

**Published:** 2026-04-21

**Authors:** Ning‐Jing Luo, Hang Chen, Meng‐Yu Li, Xin‐Tao He, Jian‐Wen Dong

**Affiliations:** ^1^ School of Physics & State Key Laboratory of Optoelectronic Materials and Technologies Sun Yat‐sen University Guangzhou China

## Abstract

Topological corner states with wavelength‐selective excitation and directional routing enable on‐chip wavelength‐dependent beam splitters (WDBSs) for multiplexing technology. In this work, we employ two types of topological corner states to implement WDBS in kagome lattice photonic crystals. A tight‐binding model incorporating both nearest‐neighbor and next‐nearest‐neighbor couplings is applied to describe the bulk and corner states in the second‐order topological photonic crystal. A 120‐degree‐turning interface is constructed that supports two types of corner states, where the type‐I corner state is even mode originating from nearest‐neighbor coupling and the type‐II state is odd mode originating from next‐nearest‐neighbor coupling. The spatial Fourier transformation of eigenstates indicates that the type‐I mode supports propagating waves along 30‐degree channel and is inhibited along the 90‐degree channel, and vice versa for type II. With different resonant wavelengths, the two types of corner states have direction‐wavelength locking mechanisms, enabling us to propose a 1 × 2 channel WDBS. Such WDBS design has achieved a low‐crosstalk multiplexing based on the directionality of corner states. This finding provides guidance for multiplexing different types of topological photonic states, which is conducive to promoting the design of on‐chip wavelength/mode‐division multiplexers with topological protection.

## Graphical abstract

Higher‐order topological corner states in kagome photonic crystals exhibit a direction‐wavelength locking mechanism: two corner states with distinct resonant frequencies couple to different output channels. Harnessing this effect, a 1 × 2 wavelength demultiplexer achieves low‐crosstalk signal separation, demonstrating the potential of topological states for integrated photonic applications.

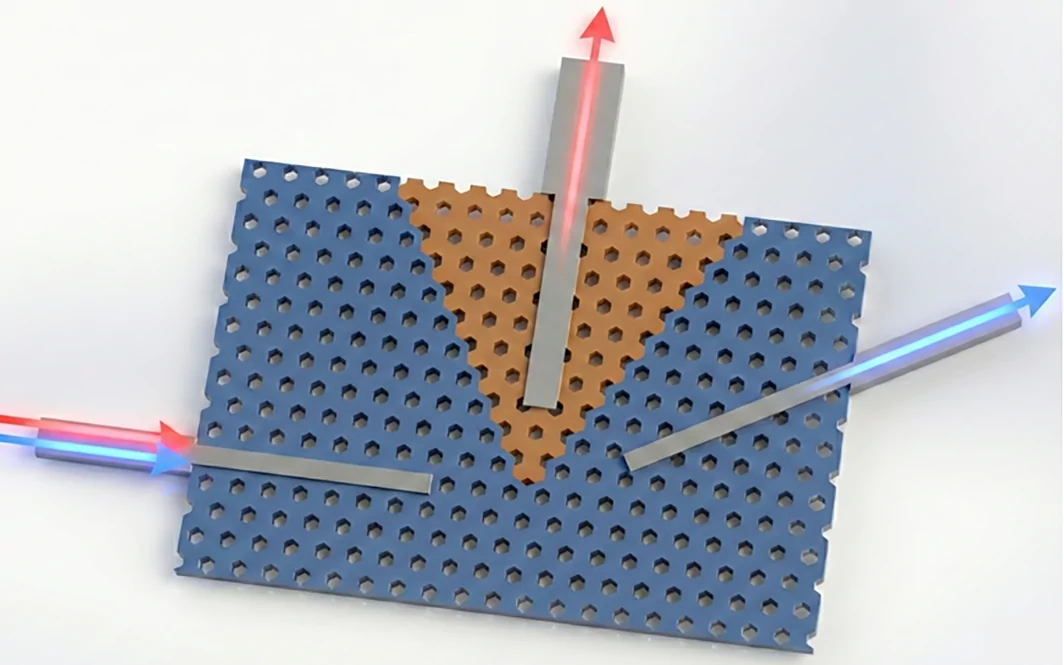

## 1 Introduction

Topological insulators have undergone extensive theoretical and experimental investigations, primarily driven by their unique edge states and exceptional transport characteristics [1, 2]. In recent years, the concept of higher‐order topological insulators has been proposed featuring lower‐dimensional boundary states, which go beyond the conventional bulk‐edge correspondence of traditional topological insulators (i.e., a d‐dimensional [dD] topological insulator possesses (d‐1)D edge states) [3, 4, 5, 6, 7]. For example, in the case of a second‐order 2D topological insulator, not only do 1D edge states propagating along the boundary exist, but also 0D corner states localized at the corners. In photonic systems, photonic crystals (PCs) have emerged as a promising platform for implementing topological concepts due to their tunable geometric structures in subwavelength units and highly controllable band dispersions [8, 9, 10, 11, 12]. Topological photonic crystals (TPCs) have been used to demonstrate many topological physics with non‐trivial phases, such as quantum Hall effect [9, 13], quantum spin Hall effect [14, 15, 16, 17], quantum valley Hall effect [18, 19, 20], and second‐order topology with nonzero 2D Zak phase [21, 22, 23, 24, 25]. One of the typical mechanisms to realize the second‐order non‐trivial topological phase is the 2D Su–Schrieffer–Heeger (SSH) model, when intra‐unit‐cell sites are connected via internal couplings and adjacent unit cells via external couplings [25, 26, 27, 28]. A topological phase transition occurs when internal and external coupling strengths are equal. The 2D photonic SSH model is more experimentally feasible: topological phase transitions can be achieved by regulating the distance between dielectric rods or air holes in the same and nearby unit cells through shrinking/expanding. The photonic second‐order corner state (SOCS) has been theoretically and experimentally verified in the square‐lattice topological PCs [25, 29, 30, 31]. Note that such a type of corner state with even symmetry is induced by short‐range interaction of nearest‐neighbor (NN) inter‐cell sites (labeled as type I). Experimental observations revealed that the type‐I corner states in PC slab exhibit a high‐Q factor, which directly verifies its potential for application as a nanocavity for intriguing light–matter interaction [32, 33, 34, 35, 36].

On‐chip optical beam splitter (BS) is one of the core components to develop multiplexing technology for integrated photonics. By independently modulating and parallel transmitting optical signals with different degrees of freedom (DoFs) of light, one can implement various types of DoF‐dependent BS (e.g., amplitude, wavelength, polarization, mode) to improve the capacity of optical information processing [37, 38, 39, 40] and also offers technical insights for metasurface research [41]. The introduction of topological properties of PCs into BS designing will be promising for realization of on‐chip multiplexing technology with topological robustness and compact size. For example, based on the first‐order TPCs with valley Hall effect, some topological BSs have been proposed for the prototype of nanodevices, including 3‐dB power splitting for quantum interference [42, 43, 44], polarization‐controllable splitting with large bandwidth [45, 46, 47] and wavelength‐dependent splitting for wavelength division multiplexing [48, 49, 50, 51]. In regard to second‐order TPC, another type of corner state (labeled as type II), dominantly induced by long‐range interaction of next‐nearest‐neighbor (NNN) inter‐cell site, has been observed in microwave kagome lattice PCs [52], and a series of works were subsequently carried out based on two types of SOCSs [53, 54, 55, 56, 57, 58]. Different from type I, the type‐II corner state manifests odd symmetry and different resonant wavelength, enabling it to manipulate two types of SOCSs simultaneously [57]. Through selectively controlling the wavelength and mode symmetry of incident waves, one can achieve a wavelength‐dependent beam splitter (WDBS) to multiplex these two types of SOCSs and can be conductive to develop the technology of topological wavelength division multiplexing. However, an intuitive understanding of the mechanism on controlling the selective excitation of corner states was previously lacking, which is limited for the practical design of topological WDBS based on the two types of SOCSs.

In this work, we realize WDBS in kagome lattice PCs by employing two types of SOCSs and revealing their direction‐wavelength locking mechanism, which provides significant guidance for the theoretical prediction of multiport topological routing. For one, the second‐order topological photonic crystals (SOTPCs) are designed by the lattice deformation of a kagome lattice PC based on a 2D SSH model. A tight‐binding model (TBM), which incorporates both NN and NNN couplings to account for long‐range interactions, is employed to characterize the bulk and corner states of SOTPCs in the kagome lattice. We construct a 120‐degree‐turning interface that supports dual corner states—exactly the two types of SOCSs, namely type I and type II. With respect to the spatial symmetry of these two states, type‐I and type‐II SOCSs correspond to an even and odd mode. This configuration can be validated through both TBM calculations and numerical simulations. Spatial Fourier transforms of these two types of SOCSs reveal a special directional selectivity that the type‐I case permits wave propagation along a 30‐degree channel while suppressing it along 90‐degrees, with the inverse behavior observed for type II. Combined with the feature of their distinct resonant wavelengths, these states exhibit direction‐wavelength locking, enabling the design of a 1 × 2 channel WDBS.

## 2 Theory of 2nd‐Order Topological Photonic Crystal

As shown in Figure 1a, the SOTPCs of our work are designed in kagome lattice. Rhombic‐profile holes (side length: *L =* 0.28*a*) are distributed on the silicon background to form a unit cell (lattice constant: *a =* 407 nm) composed of three air holes (i.e., cluster) arranged in a triangle lattice. Note that the effective permittivity of the dielectric background is *ε*_eff_ = 8.12, determined from the TE_0_ mode index of a silicon slab with a thickness of 0.541*a*. This thickness corresponds to a 220‐nm‐thick silicon slab when the operating wavelength is approximately 1550 nm. In theory, such SOTPC can be investigated by means of the 2D SSH model which provides a clear picture for analyzing its intrinsic mechanism. Figure 1e gives the schematic of a 2D SSH model consisting of intra‐cell coupling $t_{a}$, NN inter‐cell coupling $t_{b}$ and NNN inter‐cell coupling $t_{c}$. Regarding an unperturbed kagome lattice, that is the inter‐cell and NN intra‐cell couplings are equivalent, the distance from the center of the unit cell to the center of rhombic holes is $R_{1}=\sqrt{3}a/6$ (inset [a1] of Figure 1). To obtain trivial and non‐trivial second‐order topological phases, one can detune the strengths of intra‐cell coupling and NN inter‐cell coupling ($t_{a}\neq t_{b}$), corresponding to adjusting the intra‐cluster distance of three air holes in practice. By making the air holes shrink inward or expand outward, a PC (inset [a2] in Figure 1) with trivial phase can be obtained when *R* = *R*_2_ = $\frac{L}{2}$ and a PC (inset of [a3] in Figure 1) with topologically non‐trivial phase can also be obtained when *R* = *R*_3_ = $\frac{\sqrt{3}a}{3}-\frac{L}{2}$. Figure 1b–d show the TE‐polarized bulk band structures of the corresponding shrunken PC (a2), the original PC (a1) and the expanded PC (a3). For the original PC (a1), the TE‐polarized band exhibits a degenerated Dirac point at *K*. When the topological phase transitions in kagome lattice PCs are induced by expanding or shrinking the air clusters (see the inset), the degenerated Dirac point is splitting and a bandgap is opened. In this case, the trivial PC (a2) and the non‐trivial PC (a3) have the same band structure with a 15.2% band gap from 0.243c/ato0.283c/a (*c* is the velocity of light in vacuum).

**FIGURE 1:**
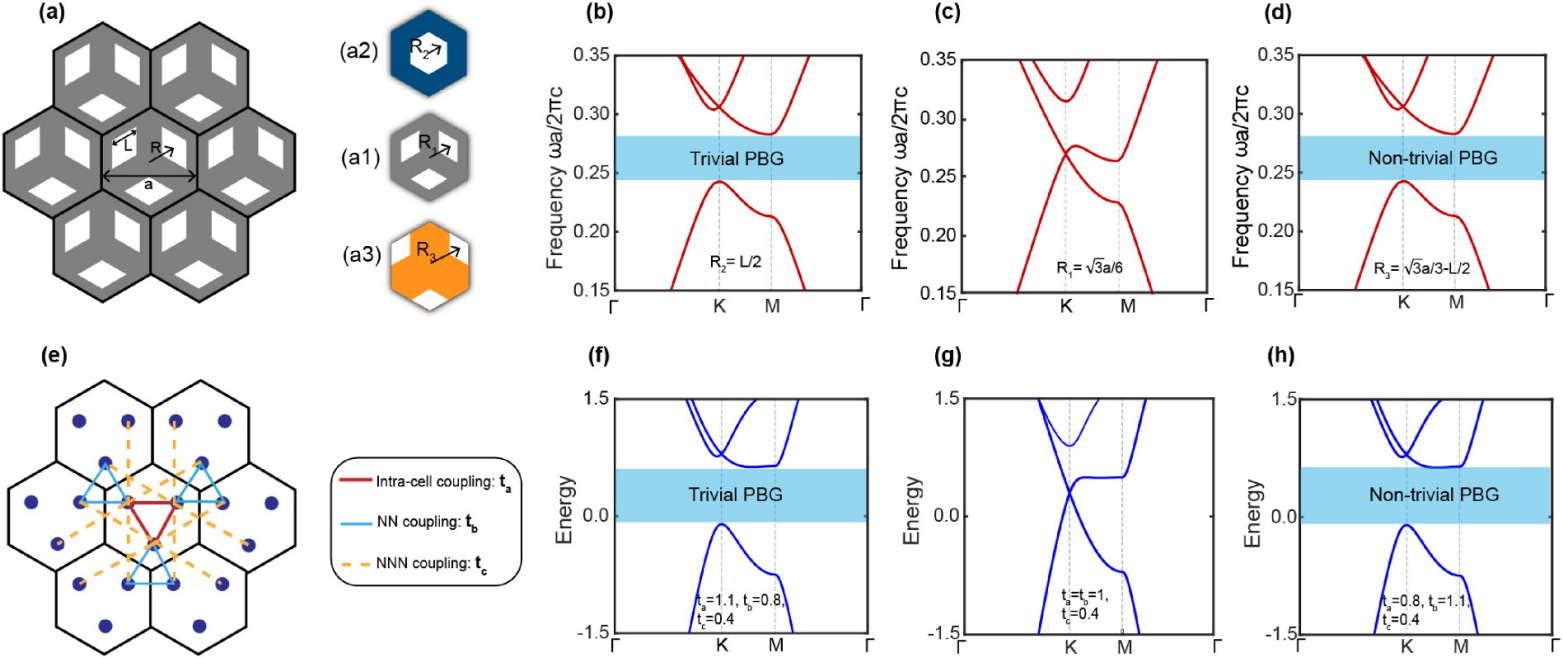
Bulk band structures of kagome lattice photonic crystals (PCs) calculated by finite‐element simulation and tight‐binding model (TBM). (a) Schematics of kagome lattice PCs composed of an array of rhombic‐profile air holes in silicon background. The hexagonal unit cell (with lattice constant *a*) contains three diamond‐profile air holes with side length *L*. *R* is the distance from the center of unit cell to the center of air holes. (b–d) TE‐polarized bulk band structures with different distance *R*. The shrunken and the expanded structure will open the degenerated points at K. When *R* = *R*_2_ or *R*_3_, a 15.2% TE‐polarized band gap (shaded in blue) is formed. (e) TBM of kagome lattice, considering intra‐cell coupling *t*_*a*_ (red solid line), nearest neighbor coupling *t*_*b*_ (blue solid line), and next nearest neighbor coupling *t*_*c*_ (orange dashed line). (f–h) Bulk band structures calculated by TBM. With finely tuning different couplings of *t*_*a*_, *t*_*b*_ and *t*_*c*_, the result is close to the PC band structures in (b–d), indicating that the TBM can approximately explain the evolution rule of band structures.

To reveal the intrinsic mechanism of topological properties and topological phase transition in theory, we will explore a TBM with proper order of inter‐cell coupling. Note that many previous works for square‐lattice SOTPCs focus on a simple TBM with only NN coupling, as it is sufficient to describe almost all of the topological properties. However, for kagome‐lattice SOTPCs, the term of NNN coupling can't be ignored to discuss more complex scenarios on corner states. Figure 1e shows the schematic of kagome lattice TBM to describe the second‐order topological PC (Figure 1a). The interaction Hamiltonian describing the model is as follows,

(1) $$H=H_{0}+H_{\text{NNN}},$$

where *H*_0_ corresponds to the intra‐cell and NN couplings Hamiltonian, and *H*_NNN_ introduces longer‐range coupling between NNN sites. Both terms of the Hamiltonian of Equation (1) exhibit the general form:

(2) $$H_{0,\text{NNN}}=-M_{i}-t\sum_{i,j}\left({\hat{a}}_{i}^{†}{\hat{b}}_{j}+h.c.\right),$$

where *t* represents different couplings as shown in Figure 1e. $\hat{a_{i}}\left({\hat{a}}_{i}^{†}\right)$ and $M_{i}$ are the creation (annihilation) operator of particles and on‐site energy term at site *i*, respectively. In momentum space, the NN coupling Hamiltonian can be expressed as,

(3) $$H_{0}=-\left(\begin{array}{ccc} M_{0} & t_{a}+t_{b}e^{i\left(-k_{x}+\sqrt{3}k_{y}\right)a/2} & t_{a}+t_{b}e^{-ik_{x}a}\\ t_{a}+t_{b}e^{i\left(k_{x}-\sqrt{3}k_{y}\right)a/2} & M_{0} & t_{a}+t_{b}e^{i\left({-k}_{x}-\sqrt{3}k_{y}\right)a/2}\\ t_{a}+t_{b}e^{ik_{x}a} & t_{a}+t_{b}e^{i\left(k_{x}+\sqrt{3}k_{y}\right)a/2} & M_{0} \end{array}\right),$$

whereas the NNN coupling term is:

(4) $$H_{\text{NNN}}=-t_{c}\left(\begin{array}{ccc} 0 & e^{i\left(k_{x}+\sqrt{3}k_{y}\right)a/2}+e^{-ik_{x}a} & e^{i\left({-k}_{x}-\sqrt{3}k_{y}\right)a/2}+e^{i\left(-k_{x}+\sqrt{3}k_{y}\right)a/2}\\ e^{i\left({-k}_{x}-\sqrt{3}k_{y}\right)a/2}+e^{ik_{x}a} & 0 & e^{i\left(k_{x}-\sqrt{3}k_{y}\right)a/2}+e^{-ik_{x}a}\\ e^{i\left(k_{x}+\sqrt{3}k_{y}\right)a/2}+e^{i\left(k_{x}-\sqrt{3}k_{y}\right)a/2} & e^{i\left(-k_{x}+\sqrt{3}k_{y}\right)a/2}+e^{ik_{x}a} & 0 \end{array}\right).$$

The coefficients *t*_*a*_ and *t*_*b*_ reflect the strengths of the intra‐ and inter‐cell couplings between the nearest air holes, which can qualitatively describe the shrinkage and expansion of three‐holes cluster in the practical PCs. The on‐site energy term for each site is the same to $M_{0}$. Compared with the simple model that only considers NN coupling, we further consider the NNN coupling *t*_*c*_ in the TBM calculations. In contrast to the fluctuation of intra‐cell and NN coupling distances, the small fluctuation of NNN coupling distance has less effect on the realistic design and experiment. Therefore, we simplify the TBM with a fixed NNN coupling coefficient *t*_*c*_. And as shown in Figure 1f–h, the TBM band structures have better agreement with those of practical PCs (Figure 1b–d), by finely adjusting *t*_*a*_, *t*_*b*_ and fixing *t*_*c*_ = 0.4. Similarly, the band structure starts from the undisturbed case with *t*_*a*_ = *t*_*b*_ = 1 that possesses degeneracy at *K* (Figure 1g), and then opens a bandgap when the intra‐cell and NN couplings are no longer equal (*t*_*a*_ ≠ *t*_*b*_). We adjust the coupling coefficients of *t*_*a*_ = 1.1, *t*_*b*_ = 0.8 and *t*_*a*_ = 0.8, *t*_*b*_ = 1.1 for the shrunken and expanded cases to feature identical bands in Figure 1f,h, which are similar to Figure 1b,d. The term of long‐range interaction (e.g., NNN coupling) is not only more precise to describe the evolution of bulk bands, but also suitable for the prediction of another type of corner states (will be discussed in the next section). Although the TBM cannot achieve precise fitting with the PC structure from a physical principle, it offers a succinct and qualitatively insightful explanation of the band structure properties. Therefore, this model is convenient to analyze the topological nature of kagome lattice PCs.

## 3 Localized Mode Based on Double Corner States

Different from the first‐order topology, higher‐order topological phases exhibit distinct characteristics beyond conventional bulk‐edge correspondence. For example, a 2D PC with a non‐trivial second‐order topological phase supports 0D corner states, that is SOCSs. In this section, we will discuss the topological properties of two types of SOCSs based on kagome lattice PCs, and analyze the frequency characteristics as *L* (the side length of the rhombic air holes) increases. As shown in Figure 2a, we designed a 120‐deg bend interface formed by trivial PC (a2) surrounding non‐trivial PC (a3) to obtain corner states. The non‐trivial PC (a3) is arranged as a triangular shape with a side length of 20*a*, and the outer region is terminated with the topologically trivial PC (a2). Due to the difference in topological phase [26, 27], there are several intrinsic states (isolated from bulk and edge states) that localize around the corner, that is, corner states. For confirmation, we obtained the eigenmodes spectrum of this 120‐deg bend interface via the finite‐element method (FEM) in Figure 2b. In simulation, the entire PC structure has a size of 85*a* × 25$\sqrt{3}$ *a* and is covered with a scattering boundary. Within the bandgap, there exist edge states and two separated eigenmodes. The red point represents type‐I corner state operating a lower frequency (around 0.263*c/a*), whereas the orange point represents the type‐II case featuring a higher frequency (around 0.264*c/a*). To gain deeper insights into the characteristics of the corner states, the evolution of eigenfrequencies is plotted as a function of side length *L* in Figure 2c, from a side length of 0.25*a* to 0.31*a*. As *L* gradually enlarges (i.e., the proportion of air in PCs increases accordingly), the eigenfrequencies of both type‐I and type‐II states increase. Particularly, the eigenfrequency curve of type I intersects with that of type II at *L* = 0.306*a*. The inset shows that the difference Δ*f* (the frequency difference between the type‐II and the type‐I SOCSs) changes from a positive value to a negative value as *L* increasing. Note that type I manifests a faster increase in eigenfrequency than that of type II, because most of the electromagnetic energy of the type‐I corner state is localized in the air‐hole region of the corner.

**FIGURE 2:**
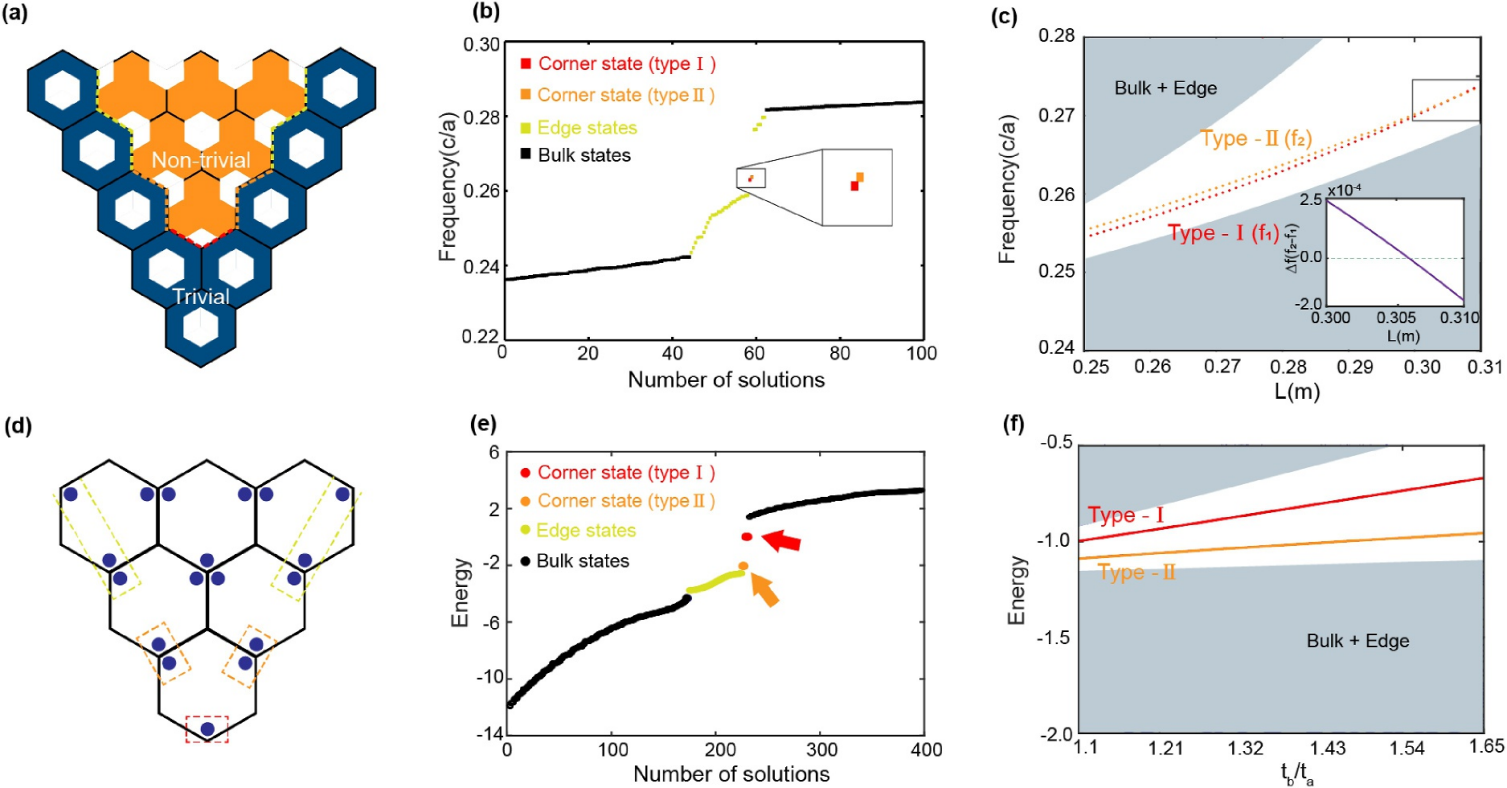
Two types of corner states in a 120‐deg bend interface. (a) Schematics of the interface formed by trivial PC (dark blue) surrounding non‐trivial PC (orange). (b) PC eigenmodes of 120‐deg bend interface. Inside the bulk band gap (shaded in blue), there are edge states (marked as yellow points) and two separated eigenmodes (marked as red and orange points) with different frequencies. (c) Eigenfrequency spectrum of PC corner states varied with the side length *L* of the diamond‐profile air hole. Inset: The frequency difference Δ*f* = *f*_2_ − *f*_1_ as a function of *L*, where *f*_1_ and *f*_2_ are the eigenfrequencies of type‐I and type‐II corner states. (d) Schematics of TBM and (e) the corresponding eigenmodes for 120‐deg bend interface. (f) Eigenenergy spectrum of TBM corner states along with ratio *t*_*b*_ */t*_*a*_ increases in a finite lattice. The NNN interaction coefficient *t*_*c*_ used in the calculations is fixed as *t*_*c*_ = 0.4 with varying ratio *t*_*b*_ */t*_*a*_, and the on‐site energy term $M_{0}$ is proportional to *t*_*a*_ ($M_{0}\propto$ *t*_*a*_). Red (for type I) and orange (for type II) lines show the results for the energies of corner states obtained with adjusting coupling coefficients.

To clarify the corner states in theory, a supercell TBM analogous to the 120‐deg bend interface is constructed. As schematically shown in Figure 2d, the corners are composed of isolated sites (marked by red dashed boxes) and dimers (marked by orange dashed boxes). The sites in the yellow dashed box represent the boundaries of the structure. For the TBM eigenmode spectrum of Figure 2e, we can observe two sets of eigenmodes (marked by red dots and orange dots) localized within the band gap and isolated from bulk and edge states, corresponding to the two types of SOCSs. The type‐II corner state is isolated from the upper bandedge of edge states (marked by yellow‐green dots). The eigenstate of type I has even‐symmetry distribution and its eigenstate energy mainly concentrates on isolated sites, whereas the eigenstate of type II (energy is dominant for dimers) has odd symmetry (see Appendix A and Figure 3). Next, we discuss the eigenenergy variation of SOCSs in TBM. An increase in *L* corresponds to a lower filling ratio of dielectric media within the unit cell for the PC structure, which will weaken the coupling between the nearest air holes and thereby reduce the intra‐cell coupling *t*_*a*_ and NN coupling *t*_*b*_. Such an effect on intra‐cell coupling is dramatically stronger than that of NN coupling, resulting in an increase in the ratio *t*_*b*_ */t*_*a*_. On the other hand, as the electric‐field energy of the corner state is in favor of concentrating on the high‐dielectric region of PC (in order for lower frequency), the decrease of filling ratio leads to reducing the energy in the dielectric region and causing an increase in $M_{0}$ (in order for higher operation frequency). Therefore, we consider the NNN interaction coefficient *t*_*c*_ used in the calculations is fixed as *t*_*c*_ = 0.4 with varying ratio *t*_*b*_ */t*_*a*_, and the on‐site energy term $M_{0}$ is proportional to *t*_*a*_ ($M_{0}\propto$ *t*_*a*_). As shown in Figure 2f, the eigenenergy of both two types of SOCSs increase when increasing the ratio *t*_*b*_/*t*_*a*_. Particularly, the eigenenergy of type I exhibits a steeper increase compared to that of type II, in agreement with the evolution trends of simulation results (Figure 2c). The inconsistencies in Figure 2 are mainly derived from the much more complex condition of PC design than TBM. For one, to precisely describe the actual PC, it is essential to include higher‐order coupling terms beyond the NN and NNN couplings in TBM. For another, the TBM interface only considers one domain wall and the inter‐cell couplings are cut off at the edge and corner, whereas the PC interface should be constructed by a topologically non‐trivial PC and another trivial PC, thereby introducing additional couplings at the edge and corner. Nevertheless, we should emphasise that the corner states in TBM maintain two key features: the deterministic existence of two types of SOCSs and their spatial symmetry, providing intuitive guidance to understand the formation mechanism of these corner states. TBM can qualitatively and intuitively describe the dispersion relationship of PCs, and clearly demonstrate the evolution process of type‐II corner state splitting from edge states through parameter scanning.

**FIGURE 3:**
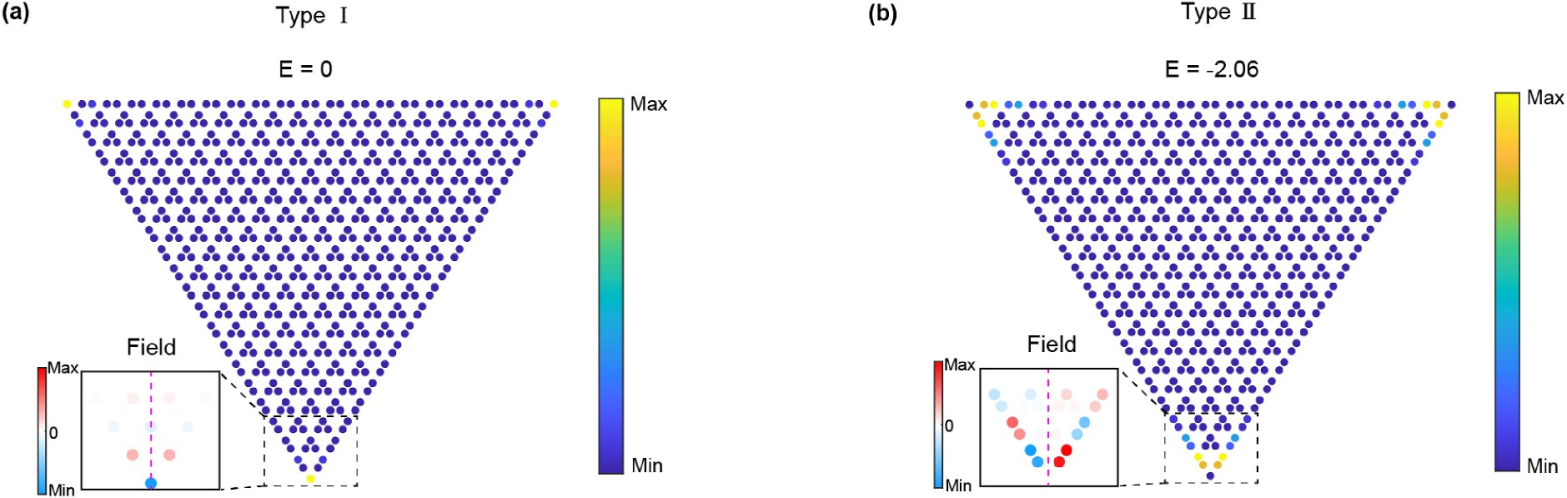
Corner states (type‐I and type‐II) distribution in TB calculation with NNN coupling. Here, *t*_*a*_ = 2/3, *t*_*b*_ = 2 and *t*_*c*_ = 0.4 in this system. (a) and (b) explicitly illustrate the energy profiles of eigenfields corresponding to the triply degenerate corner states categorized as type I and type II, respectively. Specifically, the type‐I SOCS, which is characterized by an energy of *E* = 0, exhibits a distinct spatial concentration feature that is predominantly confined to isolated lattice sites within the system. In contrast, the type‐II SOCS with an energy value of *E* = −2.06 tends to aggregate intensively on the dimer structures of the lattice. Meanwhile, the insets attached to the two figures present the real‐part distributions of eigenfields of these two types of SOCSs. Type I exhibits distinct even symmetry in their field distribution, whereas type II shows obvious odd mode characteristics in theirs.

## 4 Direction‐Wavelength Locking Mechanism

Further, we focus on the distributions of these two types of SOCSs in both the real space and momentum space, in order to reveal the direction‐wavelength locking mechanism. Figure 4a,e give the real‐part eigenfield patterns of *H*_*z*_ at resonant wavelength (*λ*_1_ = 1551 nm and *λ*_2_ = 1546 nm, respectively), that is, type‐I and type‐II SOCSs. The type‐I state exhibits an even symmetry with respect to the yellow dashed line, whereas the type‐II state is an odd mode. To further explore the resonant modes in momentum space, we extract the *H*_*z*_ eigenfields with various wave vectors by a 2D spatial Fourier transformation (FT). Figure 4b,f gives the 2D spatial FT spectrum of *H*_*z*_ for type I and type II, respectively. In the FT spectrum, the wave vector components of the excited fields are predominantly distributed along the Brillouin zone boundary, with a notable concentration at the six *K* points. This phenomenon arises from the opening of the photonic bandgap at the degenerate *K* points, which strongly modulates the propagation and localization of the excited optical modes.

**FIGURE 4:**
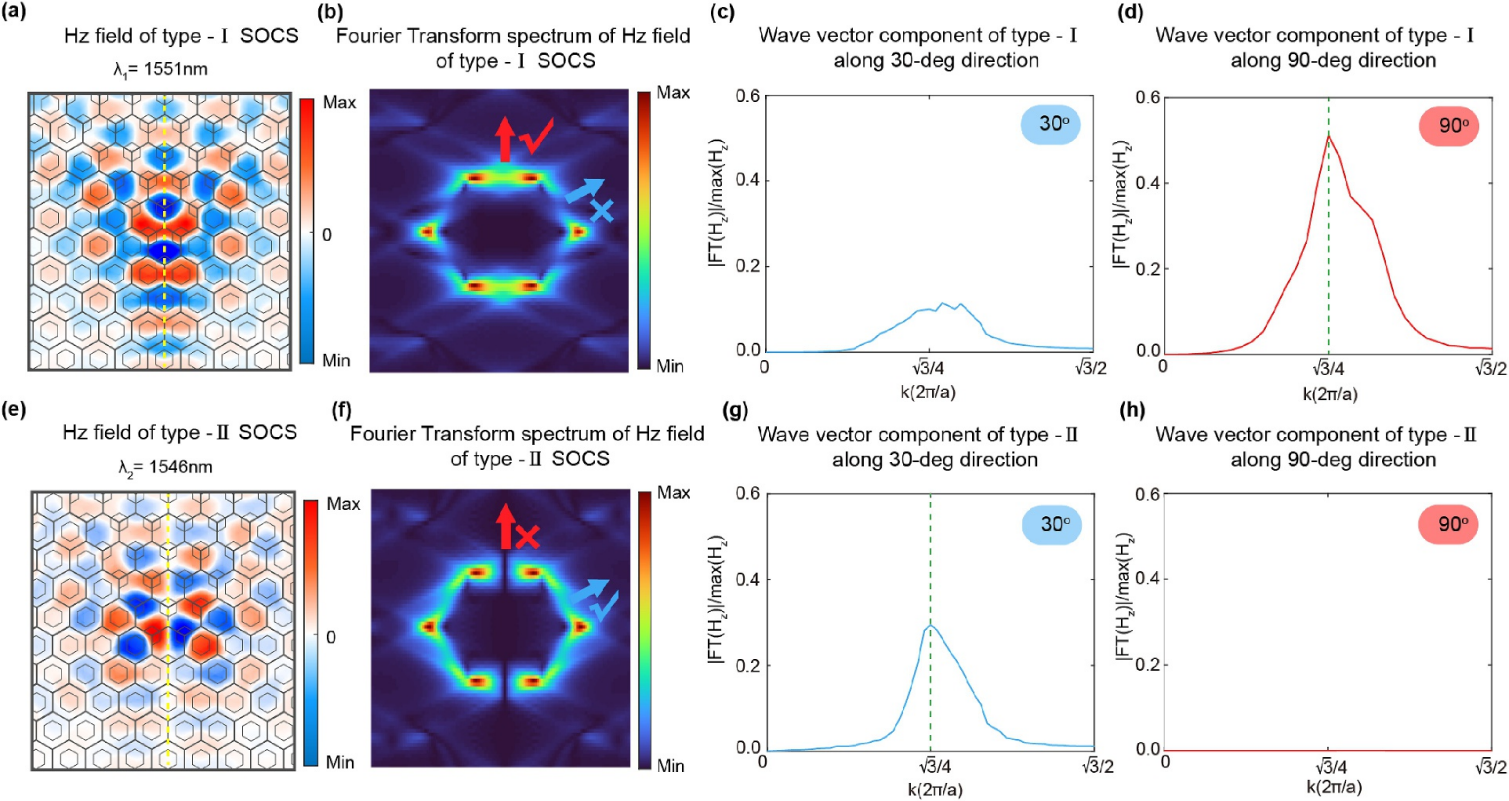
Eigenfield patterns of the two types of corner states in real space and those in Fourier space. (a) and (e) Real‐part $H_{z}$ fields in real space for type I ($\lambda_{1}=1551\;\text{nm}$) and type II ($\lambda_{2}=1546\;\text{nm}$) states, respectively. Type‐I state has even symmetry with respect to the yellow dashed line, whereas type‐II state is odd mode. (b) and (f) 2D spatial Fourier transformation (FT) spectrum of $H_{z}$ field for type I and type II, respectively. For type I, the field component along (d) 90‐deg direction is much stronger than that of (c) 30‐deg direction. On the contrary, type II presents a field component along (g) 30‐deg direction, whereas there is almost no component along (h) 90‐deg direction. That means the two types of corner states have specific directional selectivity and can be coupled to different directions, achieving spatial separation of the two states.

More importantly, the wavevector components become extremely weak in some specific directions of Γ−M, enabling us to manipulate the directional selectivity of excitation. For example, the type‐I SOCS has few components along the 30‐deg direction (blue arrow in Figure 4b) whereas type II has almost no component along 90‐deg direction (red arrow in Figure 4f). To visually demonstrate field characteristics, we also depict the distributions of wavevector components (normalized by the maximum component in *k* space) along these two directions. For Type I, the field component along the 90‐deg direction (Figure 4d) is much stronger than that of the 30‐deg direction (Figure 4c). On the contrary, Type II presents a peak at the *M* point of the 30‐deg direction (Figure 4g), whereas there is almost no component along the 90‐deg direction (Figure 4h). Due to the different resonant wavelengths of these two states, the directional selectivity is locked to wavelength, namely direction‐wavelength locking mechanism. Therefore, we can conclude that the two types of SOCSs have specific directional selectivity and can be coupled to different directions, achieving spatial separation of the two states.

## 5 Design of Wavelength‐Dependent Beam Splitter

Now we apply the directional selectivity of two types of SOCSs to design a wavelength‐dependent BS. Figure 5a shows the schematic of a 1 × 2 channel BS based on SOTPC bend interface, of which some air holes are filled with silicon to form three types of defect PC waveguides (see Appendix C and Figure 7). The footprint of designed BS is 105*a* × 28$\sqrt{3}$ *a*. In the structure, three different types of PC defect waveguides have been designed to achieve high‐efficiency coupling with three ports. The operation wavelength range of these defect modes covers the resonant wavelengths of two types of SOCSs (see Appendix B and Figure 6). The channel along 90‐deg direction (output to Port 2) supports corner mode for type I, whereas the channel along 30‐deg direction (output to Port 3) supports corner mode for type II. Line‐defect PC waveguides are introduced at all three ports with the aim of improving the coupling efficiency. In simulation, a line source is placed at the left waveguide, and a line detector (e.g. Port 2 and Port 3) is placed at the other two output waveguides to measure the transmitted power. The width of line source and line detector are the same as the width of strip waveguides. The incident waves that contain two types of SOCSs wavelengths propagate along the strip waveguide and defect waveguide, and then excite the two types of SOCSs when the coupling length between waveguide and corner is proper (see Appendix D and Figure 8 for detailed discussion of on the influence the coupling distance *l*_*3*_ of insertion loss and bandwidth). Here, *l*_1_ = 4.5*a*, *l*_2_ = 2.25 × $\frac{2a}{\sqrt{3}}$ and *l*_3_ = 6.75 × $\frac{2a}{\sqrt{3}}$. In the COMSOL simulation, the TEM mode approximation was employed to solve for these two dominant modes separately, thereby allowing the device transmittance to be calculated based on their superposition. This simplified approach accurately captures the dispersion and coupling characteristics of the waveguide while avoiding the high computational cost of a full 3D vectorial simulation, thus ensuring the reliability of the transmittance results. Figure 5b shows the simulated field distributions of the two excited wavelengths at 1556 and 1548 nm, respectively. The type‐Ι SOCSs (1556 nm) is almost guided into the defect waveguide (along $\Gamma -M^{\prime }$ direction) and output from Port 2, whereas the type‐II case is mainly guided into the defect waveguide (along Γ−M direction) and output from Port 3. To check the wavelength‐division performance, we calculate the transmittance at Port 2 (*T*_21_) and Port 3 (*T*_31_) in Figure 5c. *T*_21_ and *T*_31_ denote the ratios of the output power in Port 2 and Port 3 to the incident power in Port 1. In the transmission spectrum of the output from Port 2 (red lines), there is a dominant transmission peak at 1556 nm corresponding to type I, whereas in the transmission spectrum of the output from Port 3 (blue lines), there is a transmission peak at 1548 nm corresponding to type II. Hence, such WDBS design with 1 × 2 channel enables us to multiplex the two types of SOCSs, and can be conducive to on‐chip WDM applications based on topological modes. And we can increase the port number of output channels by cascading different designs of SOCSs, showing potential applications for multi‐beam routers in multiplexing technology. Furthermore, we have discussed the tolerance of device performance (the wavelength difference of two type of SOCSs and the device crosstalk) on the side length *L* and the slab thickness *h*, which can better establish the robustness and practicality of the proposed device (see Appendix E and Figure 9).

**FIGURE 5:**
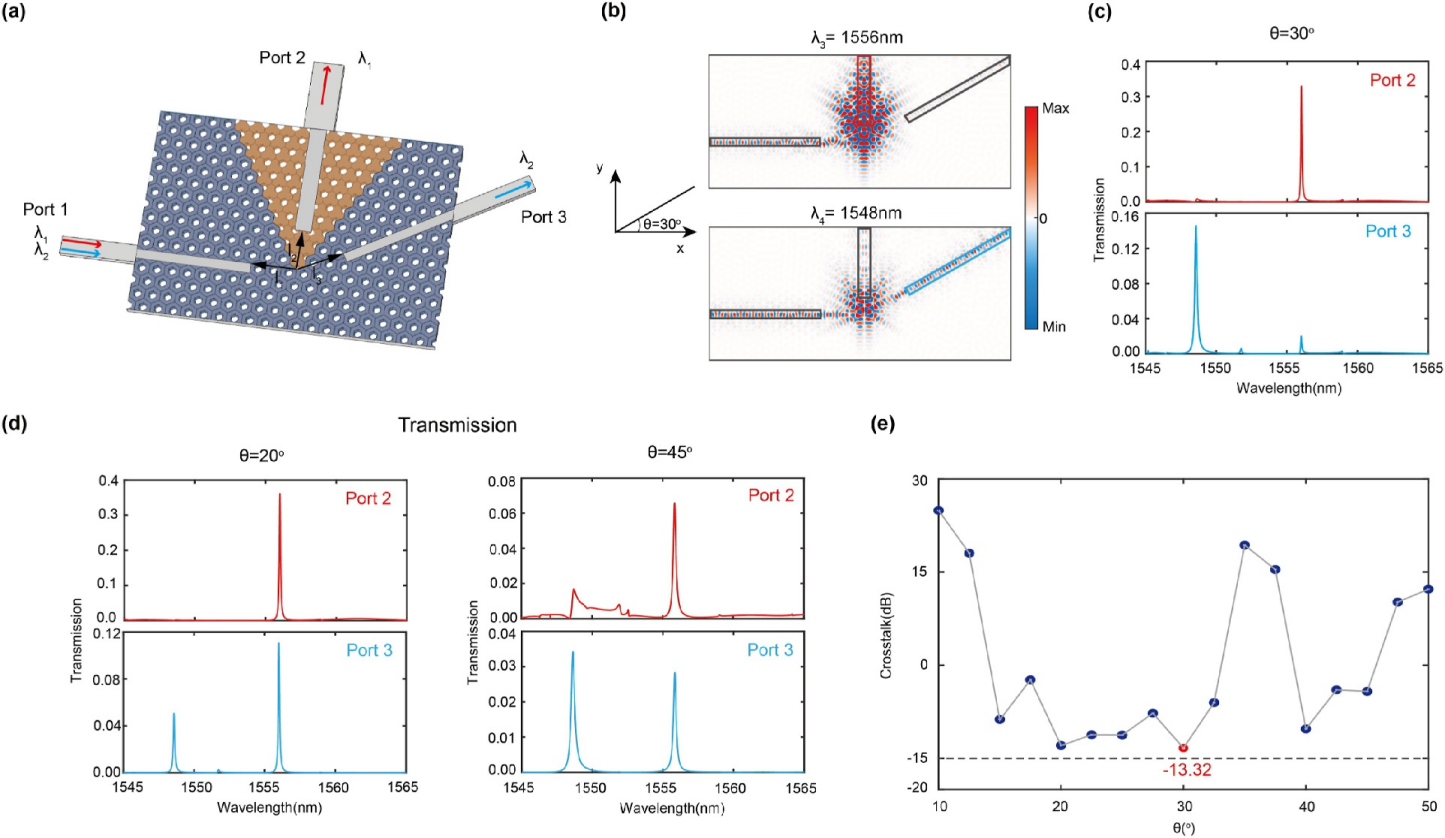
Wavelength‐dependent beam splitter. (a) Schematics of 1 × 2 channel beam splitter. Note that some air holes are filled with silicon to form a defect photonic crystal waveguide. (b) Distribution of the real‐part $H_{z}$ field at the wavelengths of 1556 and 1548 nm when the system is excited by a source placed at Port 1. (c) Simulated transmission spectra for the designed WDBS, that obtain resonant wavelength 1556 nm for Port 2 and 1548 nm for Port 3. (d) Simulated transmission spectra at *θ =* 20° and *θ =* 45°, where *θ* is the angle of orientation between Port 3 and *x* axis. (e) Crosstalk curve of Port 3 as a function of *θ*. CK is defined as the transmittance ratio of the leakage wavelength to the signal wavelength at the target port, that is $\text{CK}=10\;\mathrm{lg}\left(T_{\text{leakage}}/T_{\text{signal}}\right)$. For Port 3, we aim to selectively excite the type‐II corner state, and thus the signal (leakage) wavelength should be 1548 nm (1556 nm).

**FIGURE 6:**
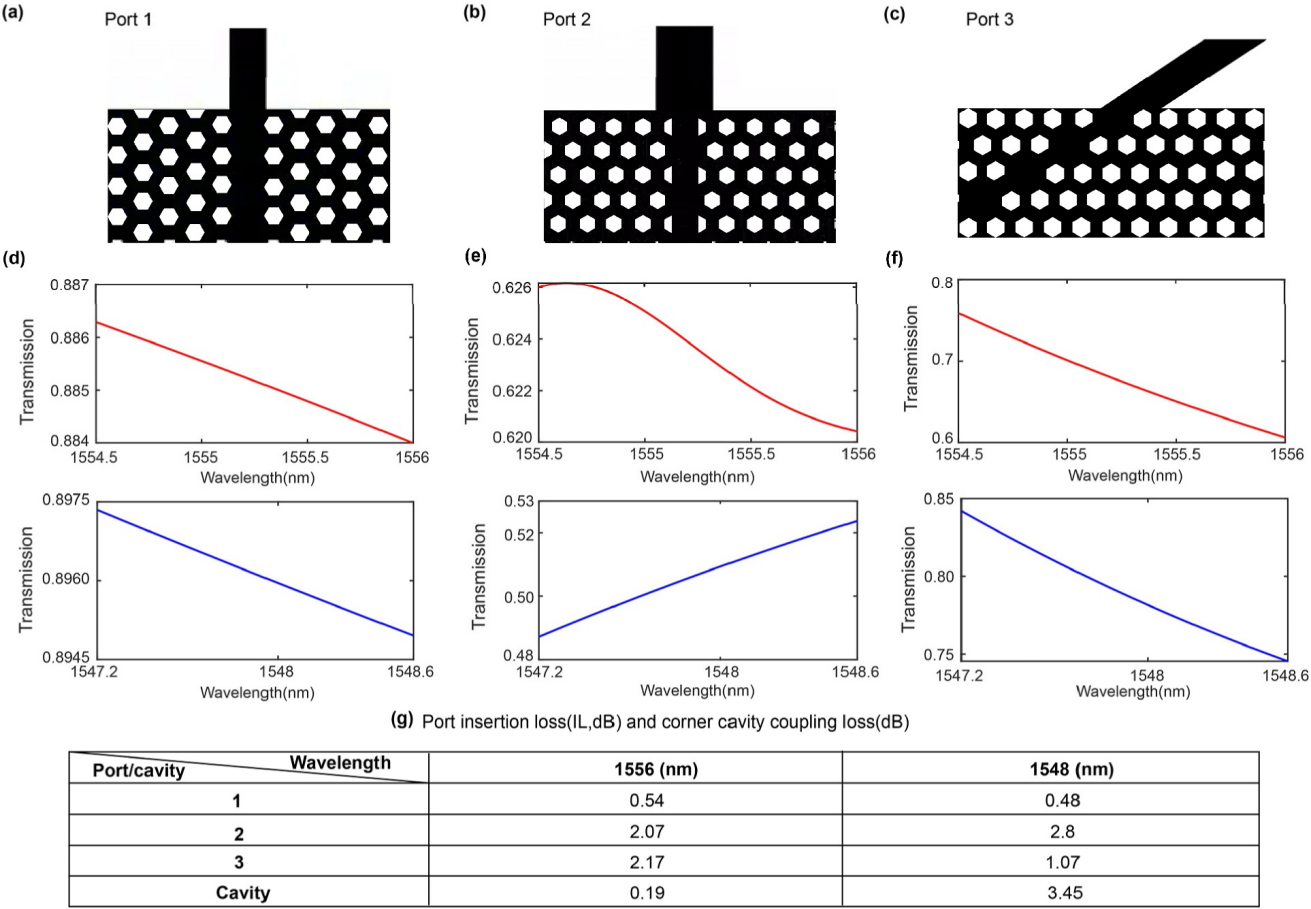
Three interface types and their corresponding insertion losses (IL) and corner resonant cavity coupling losses. (a–c) Schematics of the three types of interfaces. (d–f) The transmission spectra for each port near the two resonant wavelengths (1556 and 1548 nm). (g) The insertion losses (IL = 10log(*P*_out_/*P*_in_)) of the three transmission ports and the coupling losses between corner resonant cavity and defect waveguides.

**FIGURE 7:**
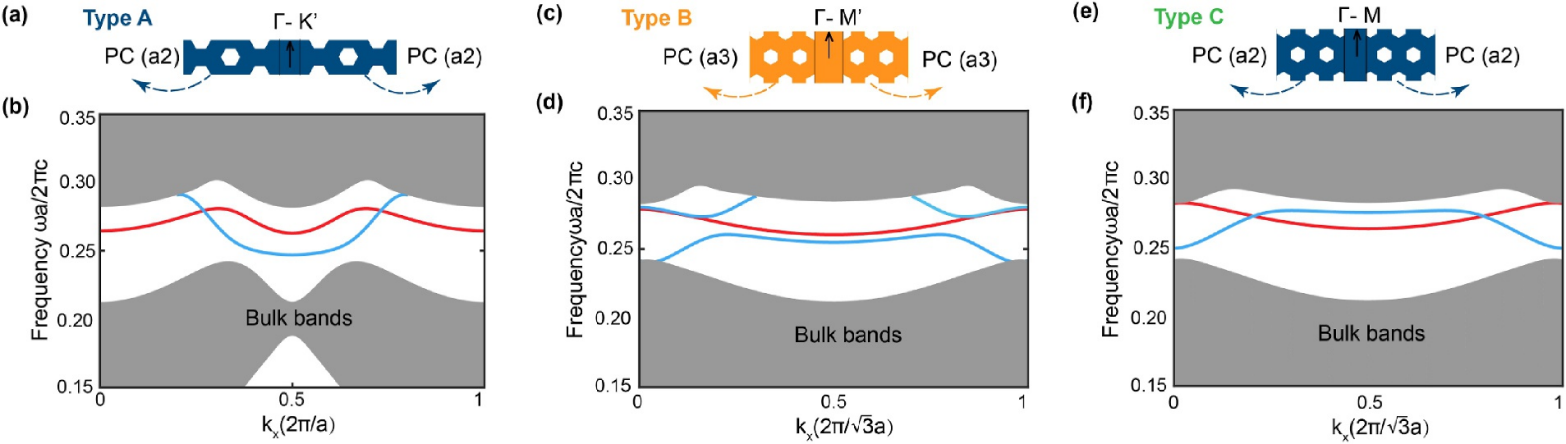
Projected band structures of the three types of defect waveguides. (a), (c) and (e) depict three types of silicon defect waveguides oriented along the $\Gamma -K^{\prime }$, $\Gamma -M^{\prime }$ and Γ−M directions, respectively. Type A and C are surrounded by PC (a2), whereas Types B is enclosed by PC (a3). And (b), (d) and (f) show the corresponding projected band structures of these waveguides. There are even modes (i.e., even modes exhibit field symmetry about the waveguide axis) depicted by the solid blue line and odd modes (show antisymmetry) depicted by the solid red line.

**FIGURE 8:**
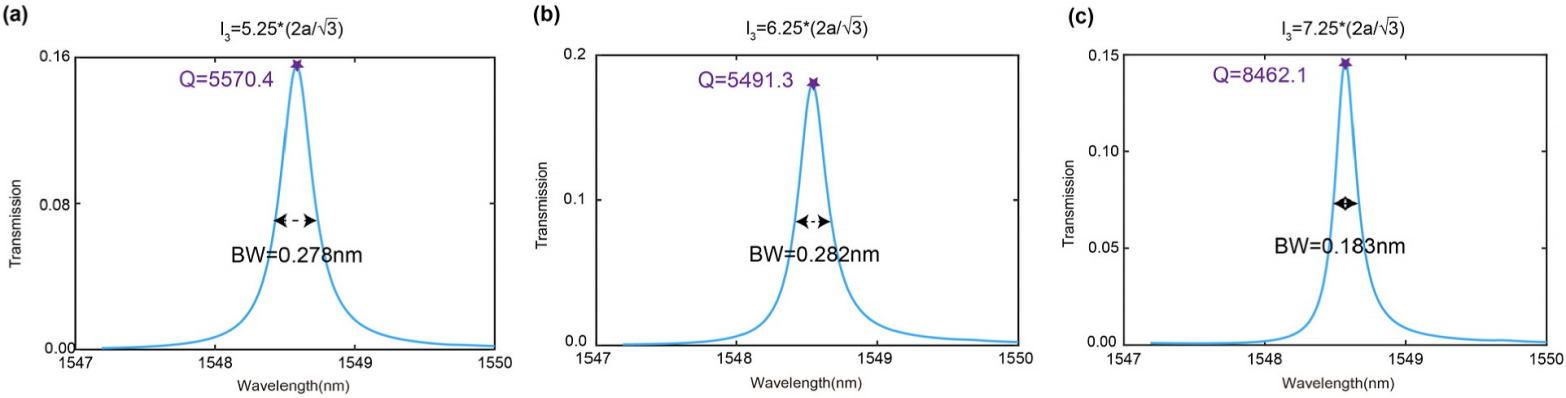
Transmission spectra of WDBS for different coupling distances *l*_*3*_. (a–c) show the transmission spectra of Port 3, along with the evolution of the corresponding bandwidth (BW) and quality (Q) factor of resonance wavelength as a function of *l*_3_, respectively. The corresponding Q factor and BW are labeled for each resonance peak, illustrating the tradeoff between Q factor and bandwidth as the coupling distance is varied.

**FIGURE 9:**
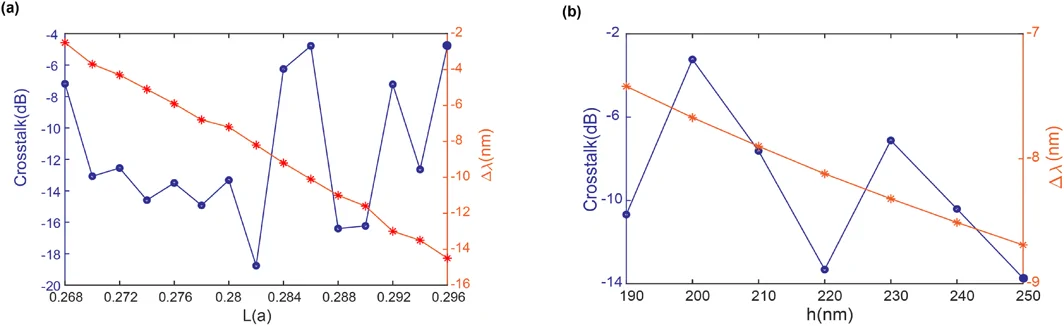
The spectrum of device crosstalk and dual‐resonance wavelength difference (Δ*λ*) on key geometric parameters (side length *L* and slab thickness *h*). Here, Δ*λ* represents the wavelength separation between the two corner states, defined as *λ*_II_ *–λ*_I_. The crosstalk (CK) is plotted as a blue solid line with filled circles, whereas the Δ*λ* is represented by a red solid line with asterisk symbols. (a) The evolution of CK and Δ*λ* with *L*. As *L* increases, the Δ*λ* gradually increases and shows a clear monotonic trend, indicating that the spectral separation of the two types of corner modes has a predictable tuning range. Specifically, when *L* ranges from 0.268*a* to 0.296*a*, the |Δ*λ|* exceeds 2 nm, enabling clear spectral separation of the dual‐resonance wavelength. The crosstalk, however, displays a more complex non‐monotonic behavior, with a range of *L* (approximately 0.27*a*–0.282*a*) in which the crosstalk remains below −10 dB. (b) CK and Δ*λ* versus *h*. As for *h*, the |Δ*λ|* still exhibits a gradual increasing trend and remains clearly separable over *h* = 190–250 nm.

Finally, we will discuss the variation of wavelength‐dependent splitting by changing angle *θ* of orientation between Port 3 and the *x* axis. Figure 5d shows the transmission spectra for *θ =* 20° and *θ =* 45° designs. Both Type‐I and Type‐II SOCSs are coupled to Port 3 that degrade the wavelength‐division performance of WDBS, although Port 2 can basically maintain selective couple of Type I. To compare the beam splitting more intuitively, we calculate the crosstalk (CK) that one of the important performance indicators for optical multiplexing devices. Here, the CK is defined as the transmittance ratio of the leakage wavelength to the signal wavelength at the target port, that is $\text{CK}=10\;\mathrm{lg}\left(T_{\text{leakage}}/T_{\text{signal}}\right)$. For Port 3, we aim to selectively excite the type‐II corner state, and thus the signal (leakage) wavelength should be 1548 nm (1556 nm). Figure 5e gives the CK curve of Port 3 as a function of *θ*. We can observe the CK curve reaches a minimum value (i.e., −13.32 dB) at *θ =* 30°, in correspondence with the FT analysis in Figure 4. For Port 3, there is another crosstalk dip at *θ* = 40° (target to couple the type‐II corner state at 1548 nm), but the type‐I corner state at 1556 nm is coupled simultaneously and thus the CK for Port 2 degrades sharply. In a word, the wavelength‐division mechanism based on the selective excitation of corner states can be theoretically predicted, and thus the two output channels separate different wavelengths deterministically. It will benefit us to design WDBS with topological robustness on dimensional bias.

## 6 Conclusion

In summary, we have theoretically explored the eigenstates of SOTPCs in kagome lattice, and discussed the eigenfrequency variation of the two types of SOCSs by changing the size length of PC structures and coupling coefficients of TBM. The two types of SOCSs with different resonant wavelength manifest distinct directional selectivity, and thus we have successfully applied the direction‐wavelength locking property to implement a topological WDBS. The manipulation of wavelength DoFs in SOCSs will enrich the capability of light manipulation in the emerging field of topological nanophotonics. The proposal of topological WDBS provides a novel method to design on‐chip passive devices, particularly for the development of wavelength/mode‐division multiplexers with topological protection. Besides, benefiting from the high‐Q modes and resonant wavelength distinction of SOCSs, we can promote the topological features of SOCSs into light–matter interaction with dual‐wavelength effect, including exciton/phonon polariton and nanolasers.

## Author Contributions

**Ning‐Jing Luo:** conceptualization, methodology, software, data curation, formal analysis, investigation, validation, visualization, writing – original draft, writing – review and editing. **Hang Chen:** software, investigation, data curation. **Meng‐Yu Li:** conceptualization, software, investigation. **Jian‐Wen Dong:** funding acquisition, project administration, supervision. **Xin‐Tao He:** conceptualization, formal analysis, funding acquisition, project administration, supervision, writing – review and editing.

## Data Availability

The data that support the findings of this study are available from the corresponding author upon reasonable request.

## Appendix A Eigenfield Distributions of Two Types of Corner States in TBM

Herein, consider intra‐cell coupling $t_{a}$, NN coupling $t_{b}$, and NNN coupling $t_{c}$, we obtain the field profile of two types of SOCSs in TBM calculation. Based on the triangular kagome lattice in Figure 1e, we construct a finite lattice (630 sites) for *t*_*a*_ = $\frac{2}{3}$, *t*_*b*_ = 2 and *t*_*c*_ = 0.4. Figure 3a,b explicitly illustrate the energy profiles of eigenfields corresponding to the triply degenerate corner states categorized as type I and type II, respectively. Specifically, the type‐I SOCS, which is characterized by an energy of *E* = 0, exhibits a distinct spatial concentration feature that is predominantly confined to isolated lattice sites within the system. In contrast, the type‐II SOCS with an energy value of *E* = −2.06 tends to aggregate intensively on the dimer structures of the lattice. Meanwhile, the insets attached to the two figures present the real‐part distributions of eigenfields for these two states. Type I exhibits distinct even symmetry in their field distribution, whereas type II shows obvious odd mode characteristics. The parity characteristics of the two types of SOCSs are consistent with the simulation results from FEM. Note that the type‐I corner state pins to the “zero energy” level (the ground frequency), which is attributed to the diagonal elements ($M_{0}$) of the Hamiltonian matrix.

## Appendix B Projected Band Structures of the Three Types of Defect Waveguides

Herein, we have performed mode analysis of the three types of defect waveguides (Type A, Type B, and Type C) involved in the coupling process. Figure 7a shows silicon defect waveguide (i.e., Type A) along the $\Gamma -K^{\prime }$ direction which correlates with the input waveguide that is shown in Figure 5a. And Figure 7c,e show a defect waveguide (i.e., Type B and C) along the $\Gamma -M^{\prime }$ and Γ−*M* direction, respectively. Specifically, the Type A and C waveguides are surrounded by PC (a2), whereas the Type B waveguide is enclosed by PC (a3). To characterize the light‐guiding behavior of these defect waveguides, we numerically simulated the three structures and the dispersion of the waveguide modes is clearly shown in Figure 7b,d,f. All three defect waveguides support at least two mirror‐symmetry‐distinct guided modes, that is one is even symmetric (blue line) and the other is odd symmetric (red line). The defect modes of Type A covers a frequency range from 0.247*c/a* to 0.291*c/a*, whereas those of Type B and Type C cover the ranges of 0.241*c/a*–0.288*c/a* and 0.25*c/a*–0.282*c/a*, respectively. The operating range of the above three types of waveguides covers the corner states (i.e., around 0.263–0.264*c/a*).

## Appendix C The Insertion Losses of the Three Interfaces and the Coupling Losses Between Corner Cavity and Defect Waveguides

Coupling loss during light transmission is also a key performance metric for WDBS devices. Accordingly, we calculated the insertion losses (IL = 10log(*P*_out_/*P*_in_)) at the three interfaces and the coupling losses from the cavity to the defect waveguides. Figure 6a–c show the schematic diagrams of the three types of waveguide interfaces, corresponding to Port 1, Port 2, and Port 3, respectively. Figure 6d–f displays the transmission spectra for each port near the two target resonant wavelengths, where the red curves correspond to 1556 nm and the blue curves correspond to 1548 nm. These spectra illustrate the transmission characteristics of each port at the targeted operating wavelengths, and their smooth, monotonic trends reflect the spectral response of the coupling between the defect waveguides and the strip waveguides. As summarized in Figure 6g, we quantified the IL for each port and the coupling losses between the corner resonant cavity and the defect waveguides at both 1556 and 1548 nm. The table provides a clear comparison of the energy loss at each interface, which is critical for evaluating the efficiency of the coupling design and guiding further optimization of the device. Noting that the 3.45 dB coupling loss for 1548 nm light (exceeding the 3 dB low‐loss threshold), we optimized the corner cavity to defect waveguide coupling distance *l*_2_ to enhance transmittance and reduce loss in this channel in the next section.

## Appendix D Evolution of WDBS Transmission Spectra, Bandwidth and Q Factor With Coupling Distance *l*_3_

In this section, the coupling distance *l*_3_ was varied to investigate its impact on the output transmission spectra, bandwidth (BW), and qualify (*Q*) factor, with the goal of identifying the optimal *l*_3_ for minimizing coupling loss and maximizing the overall performance of the device. As shown in Figure 8a–c, for Port 3, at $l_{3}=5.25\times \frac{2a}{\sqrt{3}}$, the transmission spectrum exhibits a resonance peak with a Q factor of 5570.4 and a BW of 0.278 nm. As *l*_3_ increases to $6.25\times \frac{2a}{\sqrt{3}}$, the resonance peak intensity reaches its maximum, whereas the Q factor slightly decreases to 5491.3 and the BW narrows to 0.282 nm. This indicates that $l_{3}=6.25\times \frac{2a}{\sqrt{3}}$ provides the optimal coupling strength for maximizing the output power at Port 3. When $l_{3}$ is further increased to $7.25\times \frac{2a}{\sqrt{3}}$, the Q factor rises to 8462.1 and the BW decreases to 0.183 nm, but the peak transmission intensity is reduced compared to the case at $l_{3}=6.25\times \frac{2a}{\sqrt{3}}$. At optimized ${\;l}_{3}$, the transmittance of Port 3 is improved by 0.188, indicating the coupling loss is reduced to be 2.65 dB. This value is well below the 3 dB threshold for low‐loss on‐chip photonic integration.

## Appendix E Effect of Key Geometric Parameters on Device Performance

To guide the design of low crosstalk topological WDBS, we have discussed the evolution of crosstalk and wavelength difference (Δ*λ*) with side length *L* and slab thickness *h*. Meanwhile, by fixing pores at the edge of the unit cell, the manufacturing error at distance *R* can be ignored. Figure 9 systematically characterizes the performance of topological corner states by plotting device crosstalk (CK, blue solid line with filled circles) and dual‐resonance wavelength difference (Δ*λ* = *λ*_II_ *−* *λ*_I_, red solid line with asterisks) versus two geometric parameters: *L* and *h*. In Figure 9a, as *L* increases, Δ*λ* exhibits a clear monotonic growth, confirming that the spectral separation of the two corner modes can be predictably tuned. Specifically, when *L* ranges from 0.268*a* to 0.296*a*, |Δ*λ|* exceeds 2 nm, thereby ensuring well resolved dual resonances. The crosstalk, however, displays a more complex non‐monotonic behavior, with a range of *L* (approximately 0.27*a*–0.282*a*) in which the crosstalk remains below −10 dB. As for *h*, the |Δ*λ*| still exhibits a gradual increasing trend and remains clearly separable over *h* = 190–250 nm (Figure 9b). In summary, practical design windows are identified in the *L* spaces, where both sufficient wavelength separation and low crosstalk can be simultaneously achieved. Consequently, these results provide clear, actionable guidelines for fabricating robust topological WDBS with predictable performance and high signal integrity.
